# Mitochondrial Signature in Human Monocytes and Resistance to Infection in *C. elegans* During Fumarate-Induced Innate Immune Training

**DOI:** 10.3389/fimmu.2020.01715

**Published:** 2020-08-05

**Authors:** C. Angélica Pérez-Hernández, Carina C. Kern, Egle Butkeviciute, Elizabeth McCarthy, Hazel M. Dockrell, María Maximina Bertha Moreno-Altamirano, Bruno A. Aguilar-López, Gauri Bhosale, Hongyuan Wang, David Gems, Michael R. Duchen, Steven G. Smith, Francisco Javier Sánchez-García

**Affiliations:** ^1^Laboratorio de Inmunorregulación, Departamento de Inmunología, Escuela Nacional de Ciencias Biológicas, Instituto Politécnico Nacional, Mexico City, Mexico; ^2^Institute of Healthy Ageing and Department of Genetics, Evolution and Environment, University College London, London, United Kingdom; ^3^Department of Infection Biology, London School of Hygiene and Tropical Medicine, London, United Kingdom; ^4^Department of Cell and Developmental Biology, University College London, London, United Kingdom

**Keywords:** innate immunity, immune training, mitochondria, monocytes, *C. elegans*, infection

## Abstract

Monocytes can develop immunological memory, a functional characteristic widely recognized as innate immune training, to distinguish it from memory in adaptive immune cells. Upon a secondary immune challenge, either homologous or heterologous, trained monocytes/macrophages exhibit a more robust production of pro-inflammatory cytokines, such as IL-1β, IL-6, and TNF-α, than untrained monocytes. *Candida albicans*, β-glucan, and BCG are all inducers of monocyte training and recent metabolic profiling analyses have revealed that training induction is dependent on glycolysis, glutaminolysis, and the cholesterol synthesis pathway, along with fumarate accumulation; interestingly, fumarate itself can induce training. Since fumarate is produced by the tricarboxylic acid (TCA) cycle within mitochondria, we asked whether extra-mitochondrial fumarate has an effect on mitochondrial function. Results showed that the addition of fumarate to monocytes induces mitochondrial Ca^2+^ uptake, fusion, and increased membrane potential (Δψm), while mitochondrial cristae became closer to each other, suggesting that immediate (from minutes to hours) mitochondrial activation plays a role in the induction phase of innate immune training of monocytes. To establish whether fumarate induces similar mitochondrial changes *in vivo* in a multicellular organism, effects of fumarate supplementation were tested in the nematode worm *Caenorhabditis elegans*. This induced mitochondrial fusion in both muscle and intestinal cells and also increased resistance to infection of the pharynx with *E. coli*. Together, these findings contribute to defining a mitochondrial signature associated with the induction of innate immune training by fumarate treatment, and to the understanding of whole organism infection resistance.

## Introduction

Monocytes/macrophages are amongst the cells that comprise the innate branch of the immune system and until recently, they were regarded as devoid of immunological memory. It is currently known that these cells actually possess this biological attribute, which was named “training” to differentiate it from the adaptive immune memory (1, 2). Innate immune training allows macrophages to respond better to a secondary immunological challenge, even if the primary and secondary challenges are qualitatively different (3, 4). Innate immune training of monocytes has been successfully induced with the *Mycobacterium bovis* bacillus Calmette-Guerin (BCG) vaccine (5), *Candida albicans* (6), *Candida albicans*-derived β-glucan (7, 8), and oxidized low-density lipoprotein (oxLDL) (9, 10). Innate immune training in β-glucan- or BCG-stimulated monocytes induces a metabolic shift from oxidative phosphorylation to aerobic glycolysis and inhibition of glycolysis diminishes the LPS-induced production of TNF-α and IL-6 in BCG-trained monocytes (7, 11).

Metabolic analyses have shown that in addition to glycolysis and glutaminolysis, fumarate accumulation constitutes a metabolic signature of innate immune training; moreover fumarate itself induces trained immunity, at least in part by the activation of epigenetics-regulating ezymes that lead to trimethylation of lysine 4 on histone 3 (H3K4me3) and acetylation of lysine 27 on histone 3 (H3K27Ac), linking immunometabolic activation with long-term epigenetic changes. The epigenetic program induced by fumarate partially reproduces that of β-glucan-induced training (12). There is evidence that supports the hypothesis that mitochondria lie at the heart of immunity (13–15). In this regard, mitochondria produce a number of metabolites such as fumarate and succinate, which harbor important inflammatory signaling functions (16–20) and also provide cellular and systemic homeostasis through diverse mechanisms involving metabolite-sensing, calcium signaling, mitochondrial dynamics and cristae structure, and cell-to-cell communication (21–25).

In this study we have analyzed several functional and morphological traits of mitochondria, present during the induction phase of fumarate-mediated innate immune training in human monocytes, and explored the effect of systemic exposure to fumarate on *C. elegans* mitochondria and resistance to infection.

## Materials and Methods

### Monocyte Isolation

Peripheral blood mononuclear cells (PBMCs) were isolated from healthy donors after informed consent and under Declaration of Helsinki Guidelines (Ethics committee numbers LSHTM-5520 and LSHTM-14576) by using Ficoll-paque PLUS (GE Healthcare, Chicago, IL). Thereafter, monocytes were either enriched by adherence or isolated by positive selection, using human CD14 MicroBeads (Miltenyi, Bergisch Gladbach, Germany) and suspended in RPMI-1640 medium supplemented with pyruvate, L-glutamine, non-essential amino acids, and 10% fetal calf serum.

Cells were seeded into Petri dishes (Corning Inc, NY, USA), 12-well plates (Corning), or μ-slide 8 well chambers (Ibidi, Munich, Germany) and incubated overnight at 37°C in a 5% CO_2_ atmosphere for adherence. Cells were then used for morphological and functional assays, as indicated.

### Fumarate-Induced Training

Monocytes (5 × 10^5^/well) cultured in 12-well culture plates (Corning) were supplemented with 100 μM of monomethyl fumarate (MMF) (Sigma, St. Louis, MO, USA) and incubated for 24 h at 37°C in a 5% CO_2_ atmosphere, cells were then washed with PBS and supplemented with fresh medium, as previously described (12). As a positive control for innate immune training, monocytes were incubated with heat-killed *C. albicans* (10^5^ cells/mL), instead of MMF, for 24 h, as described (7). Culture medium was replaced after 3 and 5 days of culture. At day 7, medium was replaced, cells were added with Golgistop (BD Biosciences, San Jose, CA, USA) in order to inhibit protein secretion, and cells were stimulated with 1 μg/ml LPS (Sigma, St. Louis, MO, USA) for 4 h. Cells were carefully detached by using a cell scraper (Corning) and then fixed with Cytofix/Cytoperm (BD Biosciences) and labeled with anti-CD14-APC (HCD14) (BioLegend, San Diego CA, USA), and anti-TNF-α-FITC (Mab11) moAbs (BD Biosciences).

Production of TNF-α was analyzed by flow cytometry (FACScalibur, BD Biosciences) on 10,000 events gated in the viable cell population. Cell viability was usually >80%. Mean Fluorescence Intensity (MFI) and the percentage of TNF-α-producing cells, indicative of cytokine production, were assessed by using the CellQuest software (BD Biosciences).

### Mitochondrial Dynamics and Mitochondrial Membrane Potential (Δψm)

CD14^+^ cells (4 × 10^5^ cells/well), cultured in μ-slide 8 well chambers (ibidi GmbH, Gräfelfing, Germany) were labeled with 100 nM tetramethylrhodamine methyl ester (TMRME) (Thermo Fisher Scientific, Walthman, MA, USA) for 20 min at 37°C. Cell imaging was performed in time-lapse mode (one image every 10 min for 3 h) on a Nikon Ti-E inverted microscope with Hamamatsu ORCA-Flash 4.0 Camera, driven by NIS elements version 4.6 software using a CFI Plan Apo 60x/1.4 DIC Lambda Oil objective. Images were deconvolved using NIS elements version 4.6 software. The serial images were analyzed for mitochondrial dynamics parameters (elongation, area, and interconnectivity), using Image J software (NIH, Bethesda, MD), as described (26). For Δψm assessment, the same series of images were analyzed for mean fluorescent intensity (as indicative of Δψm), using ImageJ software (NIH).

### Cytoplasmic and Mitochondrial Calcium Fluxes

Freshly isolated human monocytes were labeled with 10 μM Fluo-4/AM (Thermo Fisher Scientific), or with 10 μM Rhod-2/AM (Thermo Fisher Scientific) for 30 min at 37°C, for the assessment of cytoplasmic and mitochondrial calcium, respectively. Cells were washed with PBS and suspended in RPMI-1640 medium supplemented with 2 mM CaCl_2_ (Sigma). Base calcium concentration, as indicated by the MFI in the FL-1 channel (for Fluo-4/AM) or in the FL-2 channel (for Rhod-2/AM) was recorded for 30 s and, then, real-time calcium mobilization in response to the addition of 100 μM MMF was recorded for 3–4 min post-stimulation. At this time, ionomycin (100 ng/mL) (Sigma) was added, as a positive control of both cytoplasmic and mitochondrial calcium flux. In another set of experiments, Rhod-2/AM-loaded cells were exposed to fumarate, in the absence (no CaCl_2_ plus EGTA) or in the presence of extracellular calcium (2 mM CaCl_2_, no EGTA), in order to analyze if intracellular calcium stores are a source for mitochondrial calcium influx.

### Mitochondrial Shape and Mitochondrial Cristae Ultrastructure

PBMC-derived monocytes, cultured in Petri dishes (Corning), were treated for 3 h with 100 μM MMF, or left untreated, as a negative control. Cells were washed with Sorensen buffer and fixed for 30 min with 3% potassium permanganate in Sorensen buffer. After fixing, cells were carefully scraped off and spun down in a 15 mL tube (Corning) and then in an eppendorf tube. Cells were washed several times with Sorensen buffer. Cells were dehydrated with ethanol, embedded in EPON 812 (Electron Microscopy Sciences, Hatfield, PA, USA) and cured in an oven at 60° C for 24 h. Ultrathin sections (70 nm) were obtained, and observed with a Jeol JEM1010 electron transmission microscope, operated at 60 kV. Electron microscopy images were analyzed with the Image J software (NIH) for the assessment of morphological characteristics of mitochondria (surface area, perimeter, Feret diameter, aspect ratio, form factor, roundness), as well as cristae ultrastructure, as described (27, 28).

### Mitochondrial Dynamics and Pharyngeal Infection in Fumarate-Treated *C. elegans* as an Infection Model

*C. elegans* maintenance was performed using standard protocols (29), with worms grown at 20 °C on nematode growth media (NGM) that was seeded with *E. coli* OP50 to provide a food source. *C. elegans* strains included: SJ4103 *zcIs14 [myo-3::GFP(mit)]* (GFP-expressed in mitochondria in muscle cells) and SJ4143 *zcIs17 [ges-1::GFP(mit)]* (GFP-expressed in mitochondria in intestinal cells). An N2 hermaphrodite stock recently obtained from the *Caenorhabditis* Genetics Center was used as wild type. From the L4 (fourth larval) stage of adulthood, animals were treated throughout life with 10, 50, or 100 μM MMF solubilized in MilliQ water, or MilliQ water alone as a negative control, which was added topically to NGM plates.

For assessing mitochondrial dynamics, after 24 h from the L4 stage, at day 1 of adulthood, live animals were mounted onto 2% agar pads under a cover slip and anesthetized by placing them in a drop of 0.2% levamisole and then immediately imaged for mitochondrial analysis. Confocal microscopy analysis was performed on a Zeiss LSM510 confocal microscope with a Plan-Apochromat 63x/1.4 Ph3 objective. Mitochondrial dynamics were assessed by measuring elongation, area, and connectivity, as described (26), using Zen Software (Zeiss).

Mortality associated with pharyngeal infection was assessed daily from the L4 stage throughout worm lifespan in order to be able to perform necropsy prior to corpse decomposition. Pharyngeal swelling was analyzed as described by Zhao et al. (30). As an additional control carbenicillin was added topically onto a 2-day-old bacterial lawn at a final concentration of 4 mM to prevent bacterial growth and worms were grown on these plates and pharyngeal infection mortality assessed.

Differential Interference Contrast (DIC) images of pharyngeal infection with *E. coli* OP50 were captured using a Zeiss Axioskop at x63 magnification on day 13 of adulthood, when pharyngeal infection is common, in order to illustrate the observable contrast between healthy, uninfected and infected worms.

### Statistical Analyses

Mitochondria shape descriptors and size measurements, as well as mitochondria cristae density and incident angles were analyzed by Wilcoxon Rank Test. Mitochondrial dynamics were analyzed by Wilcoxon Rank Test and ANOVA and Tukey's *post-hoc* test, and *C. elegans* pharyngeal infection was analyzed by ANOVA, and unpaired Student's *t*-tests. All analyses were preformed using Graph Pad Prism Software (Graphpad, La Jolla, CA). A significant statistical difference between controls and treatments was defined as *p* < 0.05.

## Results

### Fumarate-Treated Monocytes Produce More TNF-α in Response to LPS Than Non-stimulated Monocytes

In order to set up the experimental conditions for the induction of innate immune training with fumarate, as reported by Arts et al. (12), monocytes were supplemented with 100 μM MMF, rested for 7 days and then stimulated with LPS for the assessment of TNF-α production. Non-trained monocytes (medium alone) and monocytes that were supplemented with a preparation of heat-killed *C. albicans*, as reported by Quintin et al. (6), served as controls.

**Figure 1:**
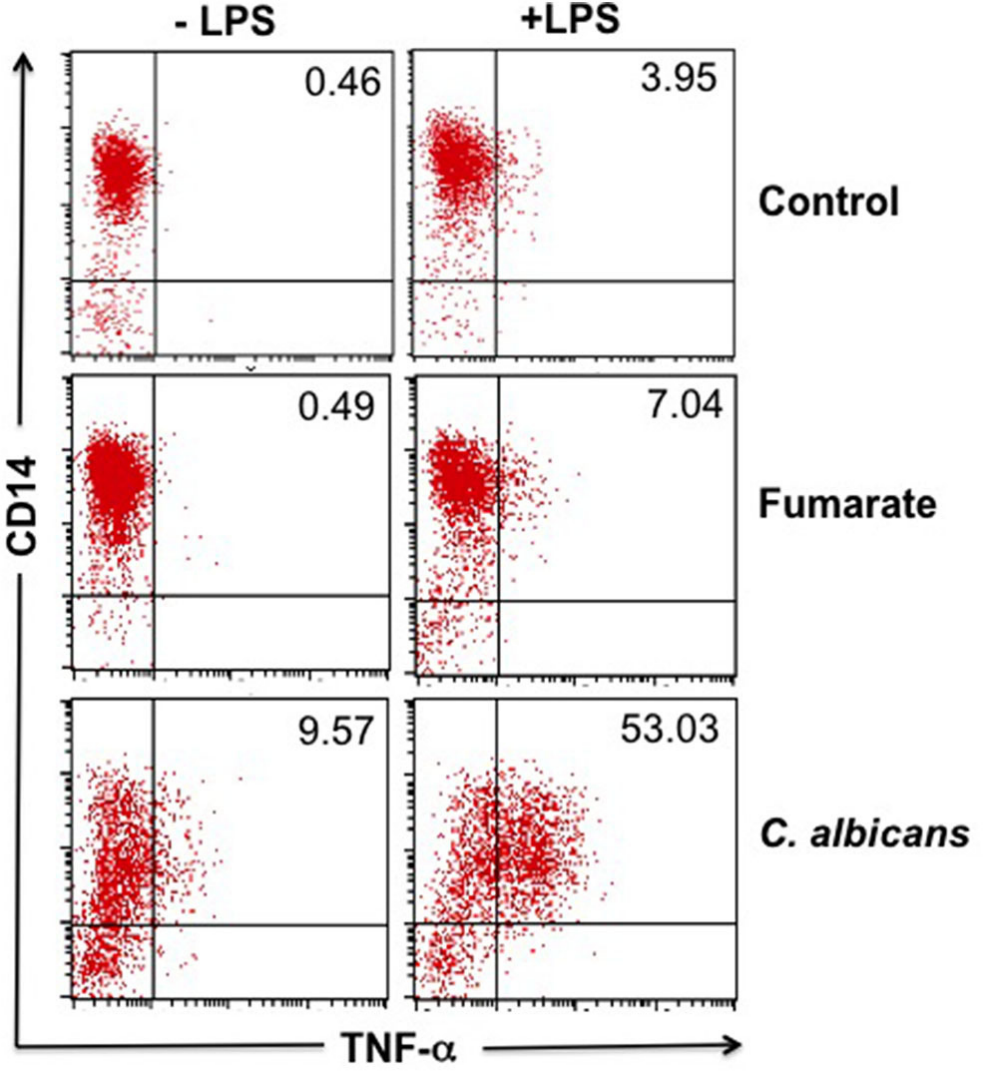
Fumarate-trained monocytes/macrophages produce more TNF-α in response to LPS than un-trained cells. Freshly isolated monocytes were trained with 100 μM MMF or with 5 × 10^6^ heat-killed *C. albicans* (as positive control) for 24 h, or left untreated (as negative control). After 7 days of “resting,” cells were stimulated with LPS for 4 h. Figure shows a representative result out of six independent experiments, showing the percentage of TNF-α-producing cells (intracellular staining), upon LPS-activation.

Results showed that, indeed, fumarate pre-stimulation renders macrophages more reactive to LPS stimulation, as assessed by the production of TNF-α, a condition compatible with innate immune training. Nevertheless, macrophages that had been pre-activated with heat-killed *C. albicans* had a higher base level, as well as a higher post-LPS level of TNF-α production than fumarate-treated cells (Figure 1).

### Fumarate Induces Cytoplasmic and Mitochondrial Calcium Uptake Within Minutes of Monocyte Stimulation

**Figure 2:**
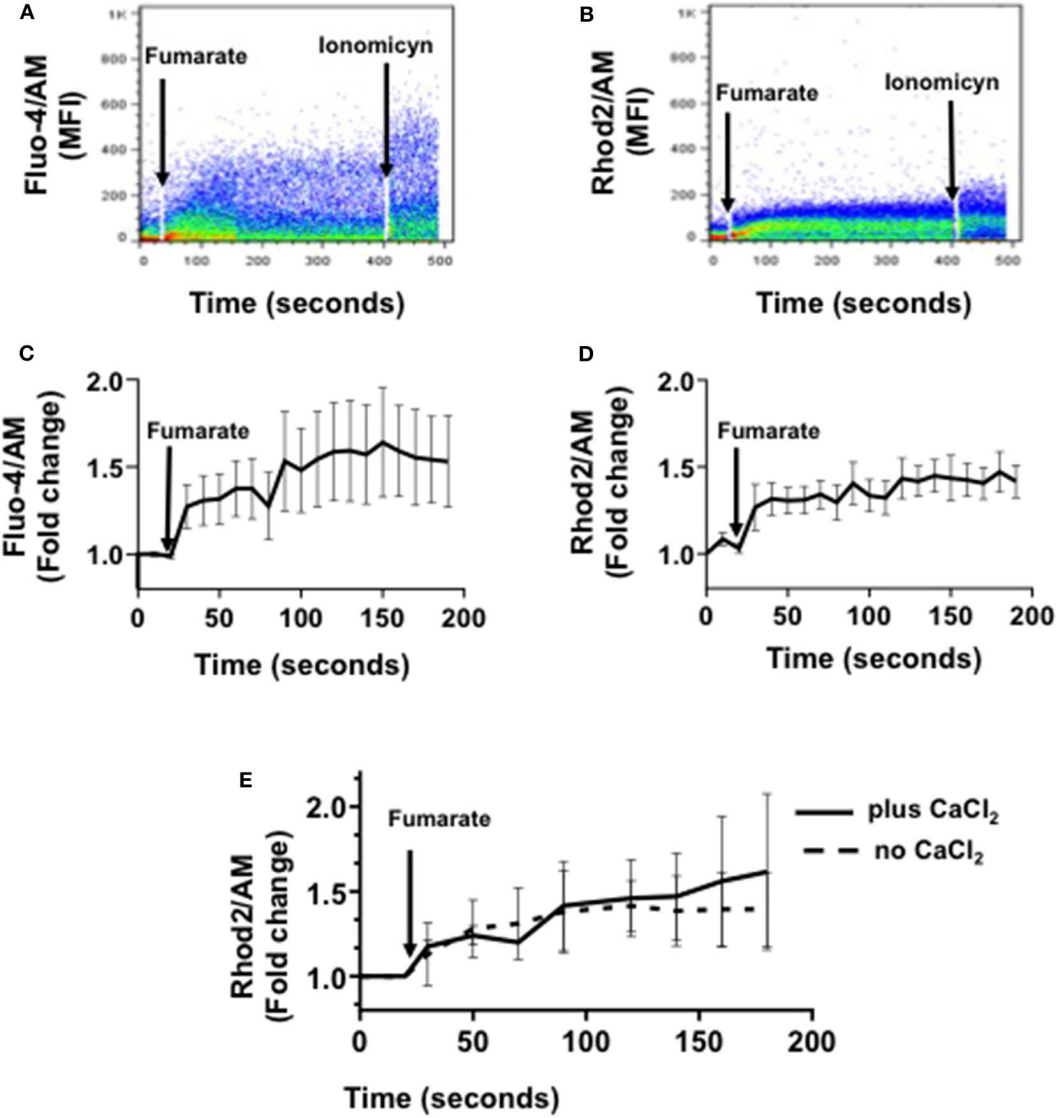
Fumarate treatment induces cytoplasmic and mitochondrial calcium influx in monocytes. Freshly isolated monocytes were loaded with Fluo-4 (cytoplasmic calcium) or with Rhod-2 (mitochondrial calcium). Mean fluorescence intensity (MFI), indicative of calcium concentration, was assessed by real time flow cytometry. After recording base calcium levels for 30 s, MMF (100 μM) was added to cells in suspension, and calcium levels were recorded, in real time, for the next 3 min. Figures depict cytoplasmic calcium influx **(A,C)**, and mitochondrial calcium influx **(B,D)**; **(A,B)** are examples of raw data, and **(C,D)** represent the mean ± s.d of the fold change in MFI (*n* = 8). **(E)** shows the kinetics of mitochondrial calcium influx in the absence, or in the presence of extracellular calcium.

After confirming that MMF readily induces innate immune training, as reported (12), we assessed a series of mitochondrial functional and morphological parameters, in order to figure out the role of mitochondria if any, in the induction phase of training, beyond that of fumarate production. Calcium flux analyses showed that within minutes of MMF addition (100 μM) both cytoplasmic and mitochondrial calcium concentrations in monocytes increased, reaching a maximal post-stimuli level at around 3 min for cytoplasmic Ca^2+^, and 2.5 min for mitochondrial Ca^2+^ (Figures 2A–D, respectively). When mitochondrial calcium was assessed in the absence or in the presence of extracellular calcium (2 mM CaCl_2_), the kinetics of calcium fluxes was similar (Figure 2E).

### Fumarate Induces Mitochondrial Fusion and Increases Mitochondrial Membrane Potential (Δψm) Within Hours of Monocyte Stimulation

**Figure 3:**
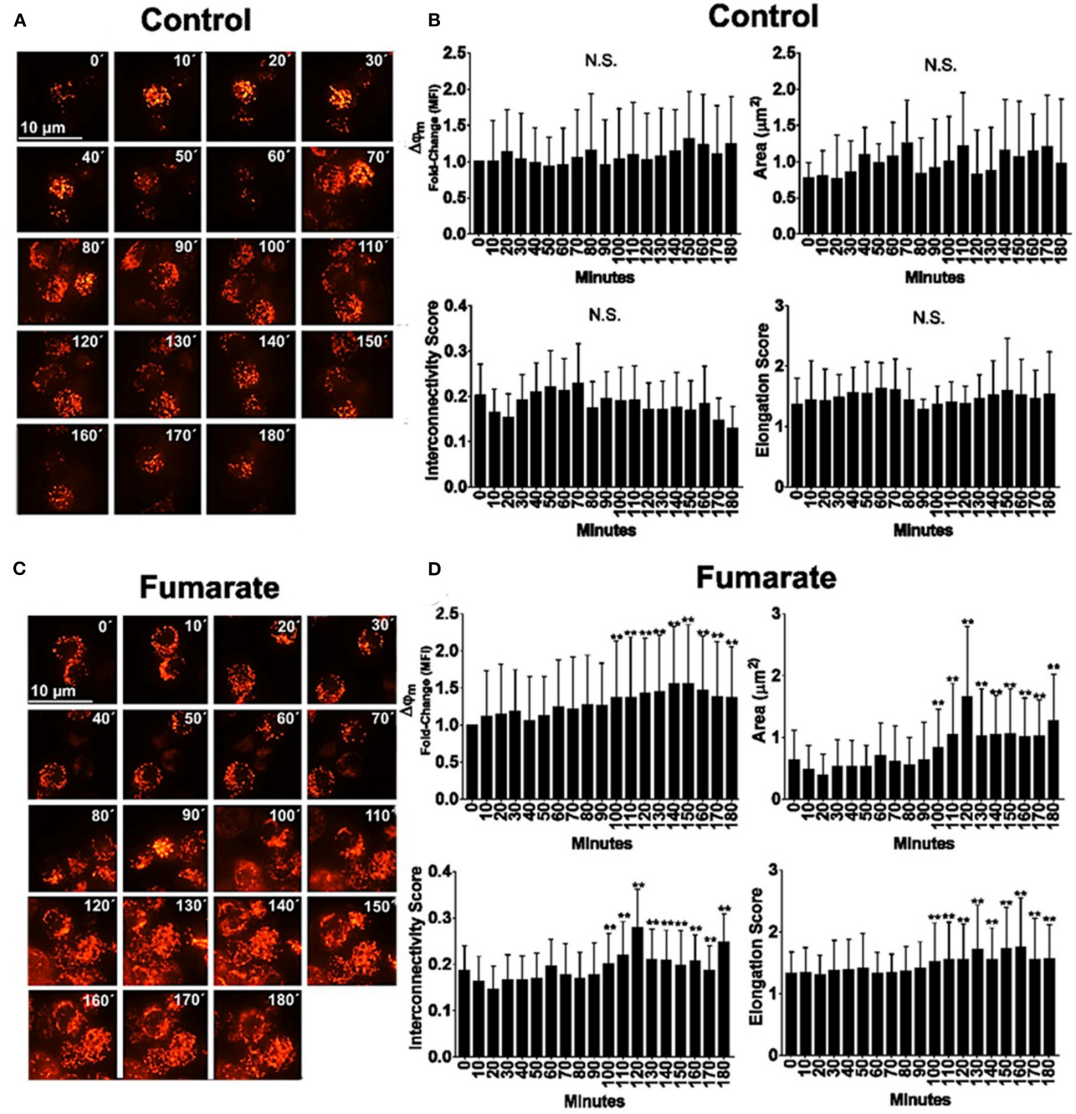
Fumarate treatment induces mitochondrial polarization and mitochondrial fusion in monocytes. Monocytes, cultured in chamber slides, were loaded with TMRME. Cells were left un-treated (medium) or supplemented with 100 μM MMF. Fluorescent images were recorded by time-lapse confocal microscopy, every 10 min, up to 180 min. Mean fluorescent intensity is indicative of Δψm, and mitochondrial dynamics parameters were assessed as indicated under Materials and Methods. Images depict raw data for medium (control) and fumarate-treated cells **(A,C)**, and figures **(B,D)** integrate the results from at least 200 individual cells per treatment, from three independent experiments. **p < 0.01 by one-way analysis of variance, Tukey *post-hoc* test.

Tetramethylrhodamine methyl ester (TMRME)-labeled monocytes were subjected to confocal microscopy analysis, for the assessment of both mitochondrial dynamics and Δψm. Results showed that, after 90 min of fumarate stimulation cells underwent mitochondrial fusion, as assessed by an increase in mitochondrial area, elongation, and interconnectivity; concomitantly, Δψm increased (Figure 3).

### Fumarate-Treatment of Monocytes Induces Changes in Mitochondrial Shape and Cristae Ultrastructure

**Figure 4:**
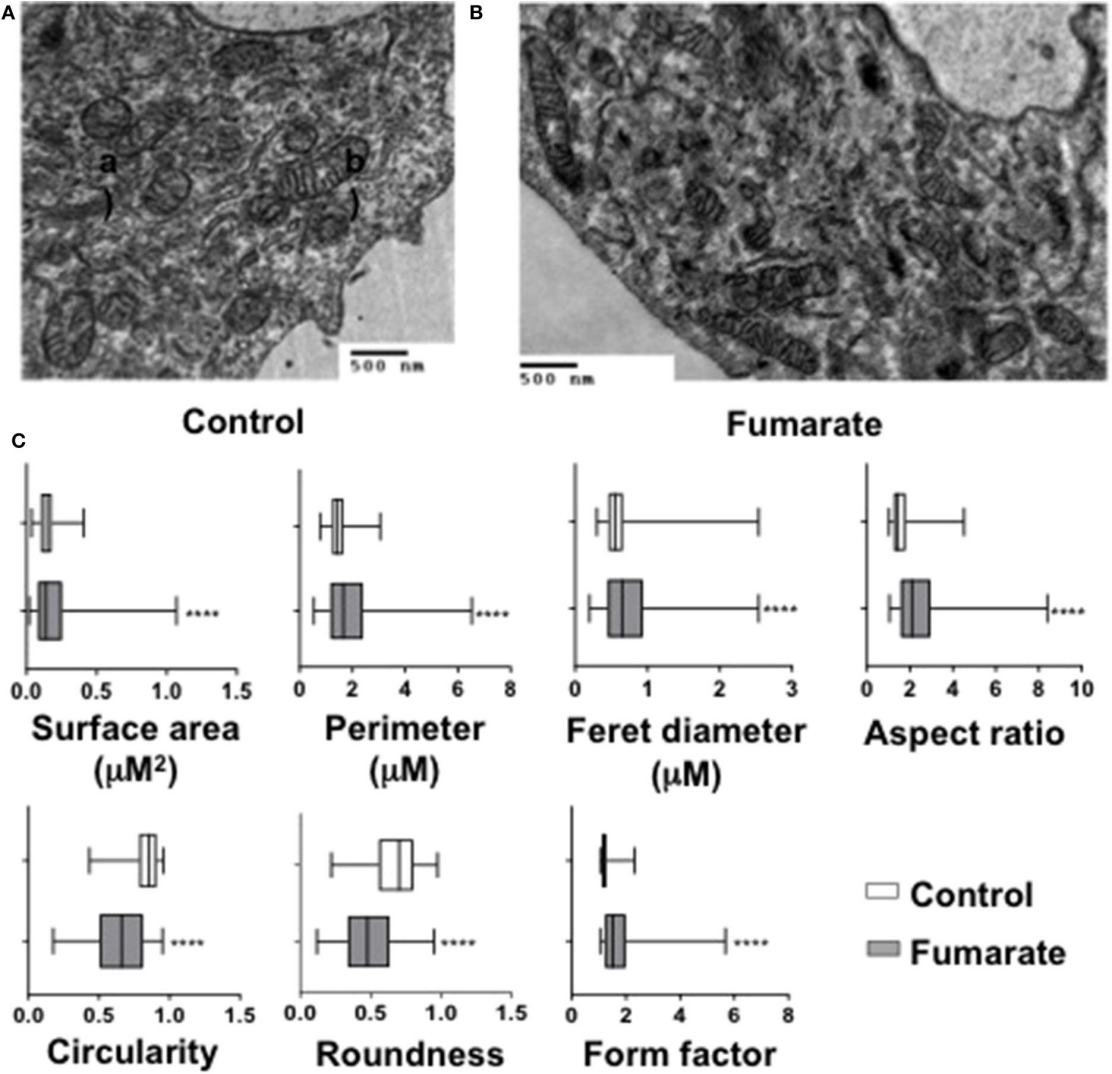
Exogenous fumarate induces changes in size and shape of mitochondria in monocytes. Freshly isolated monocytes were **(A)** left un-treated (control) or **(B)** incubated for 3 h in the presence of 100 μM MMF. Cells were then prepared for transmission electron microscopy. Ultramicroscopy images were recorded and **(C)** morphological features of mitochondria (area, perimeter, Feret diameter, aspect ratio, form factor, roundness, and circularity), were assessed as indicated under Materials and Methods. Bars indicate the range that contains 50 % of all data, middle lines represent the median, and whiskers extend toward minimum and maximum values. Results are from at least 600 mitochondria from three independent experiments. ****p < 0.0001 by Wilcoxon Rank Test, Tukey *post-hoc* test.

Transmission electron microscopy analyses were performed on MMF-stimulated monocytes/macrophages, as well as on non-stimulated cells (negative control). Results showed that upon fumarate stimulation (for 3 h) mitochondria increased their surface area (*p* < 0.0001), perimeter (*p* < 0.0001), Feret diameter (*p* < 0.01), aspect ratio (*p* < 0.0001), and form factor (*p* < 0.0001). In contrast, mitochondria from fumarate-treated monocytes had lower values for roundness (*p* < 0.001), and circularity (*p* < 0.001) (Figure 4).

**Figure 5:**
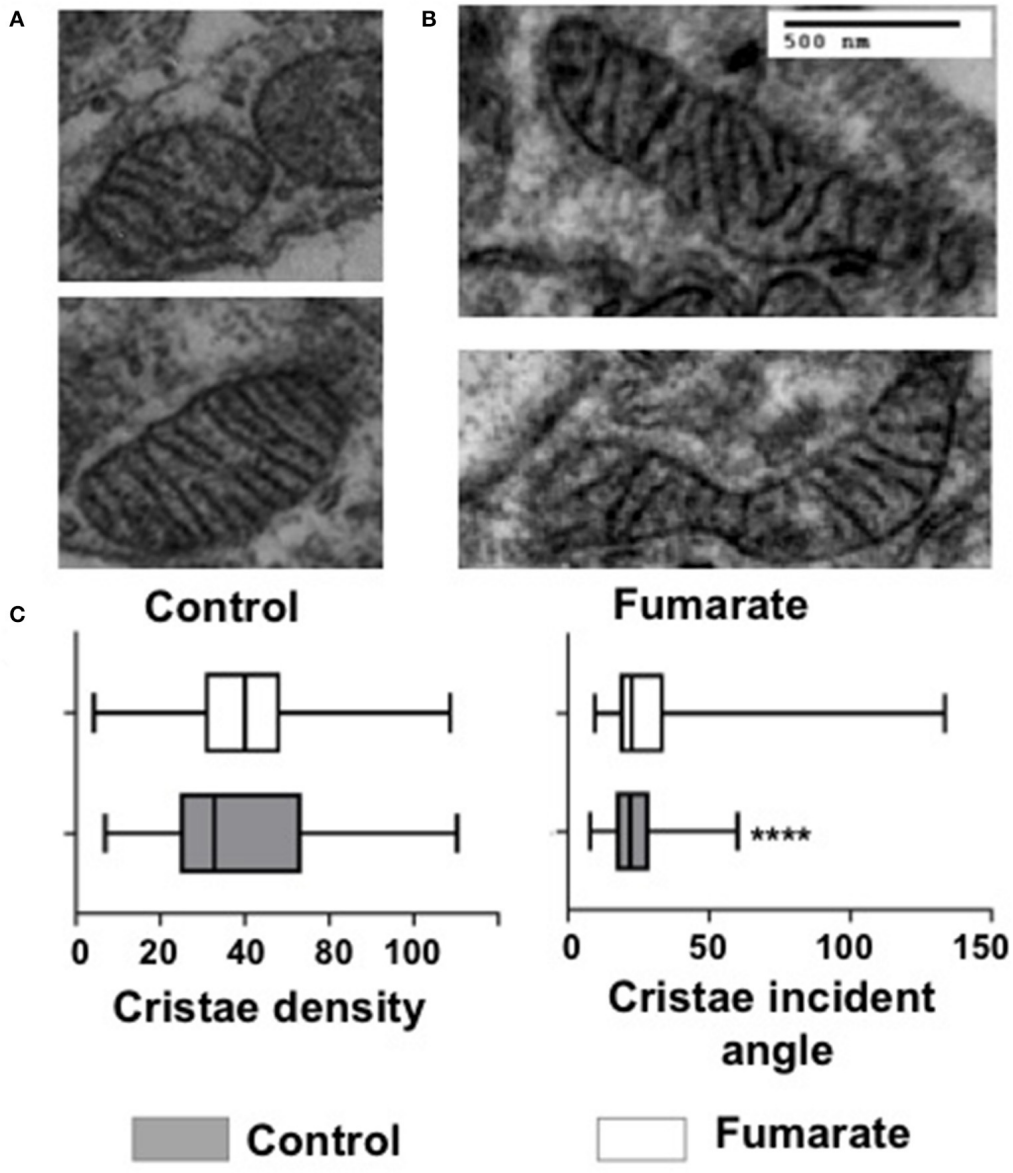
Fumarate treatment reduces the incident angle in mitochondrial cristae in monocytes. Untreated monocytes (control) **(A)** and monocytes treated with 100 μM MMF for 3 h **(B)**, were processed for transmission electron microscopy. Images were recorded and mitochondrial cristae density and cristae incident angle were calculated **(C)**, as indicated under Materials and Methods. More than 600 mitochondria from three independent experiments were analyzed. ****p < 0.0001 Wilcoxon Rank Test, Tukey *post-hoc* test.

Mitochondria cristae from fumarate-treated monocytes showed significant lower incident angles (p < 0.01) and no significant changes in cristae density (Figure 5).

### Fumarate Induces Mitochondrial Fusion in Muscle and Intestinal Cells in *C. elegans*

**Figure 6:**
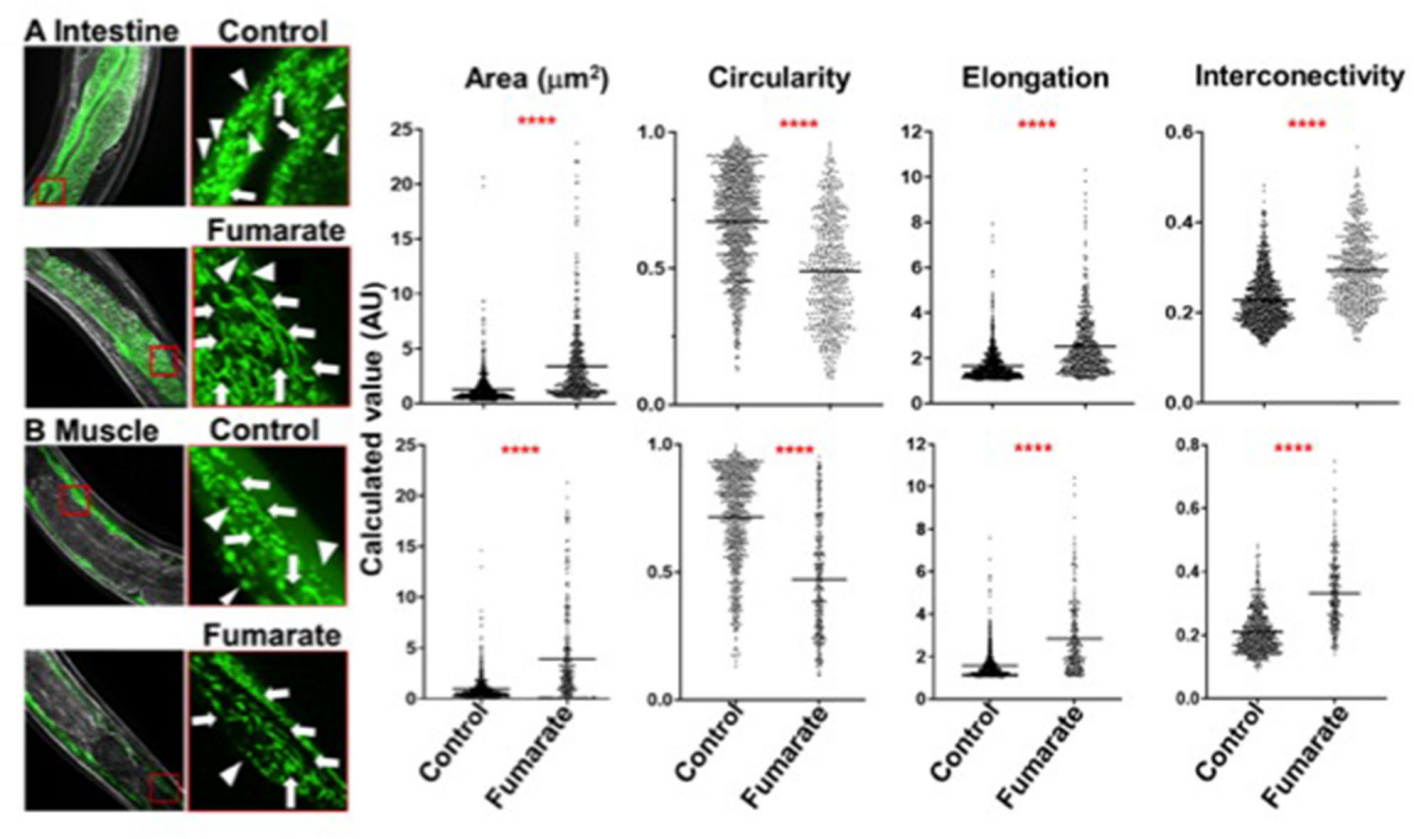
Fumarate induces mitochondrial fusion in muscle and intestinal cells of *C. elegans*. Images depict the mitochondrial dynamics of un-treated (control) and fumarate-treated *C. elegans*. **(A)** strain SJ4143 (GFP-expressing mitochondria in intestinal cells) and **(B)** strain SJ4103 (GFP-expressing mitochondria in muscle cells). Mitochondrial dynamics parameters were assessed by measuring all mitochondria within 5 squares of 30 × 30 μm, evenly distributed along the nematode, for each experimental condition. Arrow heads point to mitochondria in a fusion state, triangles point to mitochondria in a fission state, ****p < 0.0001 Wilcoxon Rank Test, Tukey *post-hoc* test (*n* = 5).

In order to analyze the effect of fumarate *in vivo* in a whole organism, *C. elegans* strains SJ4103 and SJ4143, which express GFP in muscle and intestinal cells, respectively, were exposed to 100 μM MMF just prior to adulthood at the L4 stage of development (to preclude any possible developmental effects that might confound results). After 24 h, on day 1 of adulthood, mitochondrial fusion was seen in both muscle and intestinal treated cells (Figure 6), with reduced mitochondrial circularity as well as increased area, interconnectivity and elongation, similar to changes seen in fumarate-treated human monocytes.

### Fumarate Reduces Pharyngeal Infection in Aging *C. elegans*

**Figure 7:**
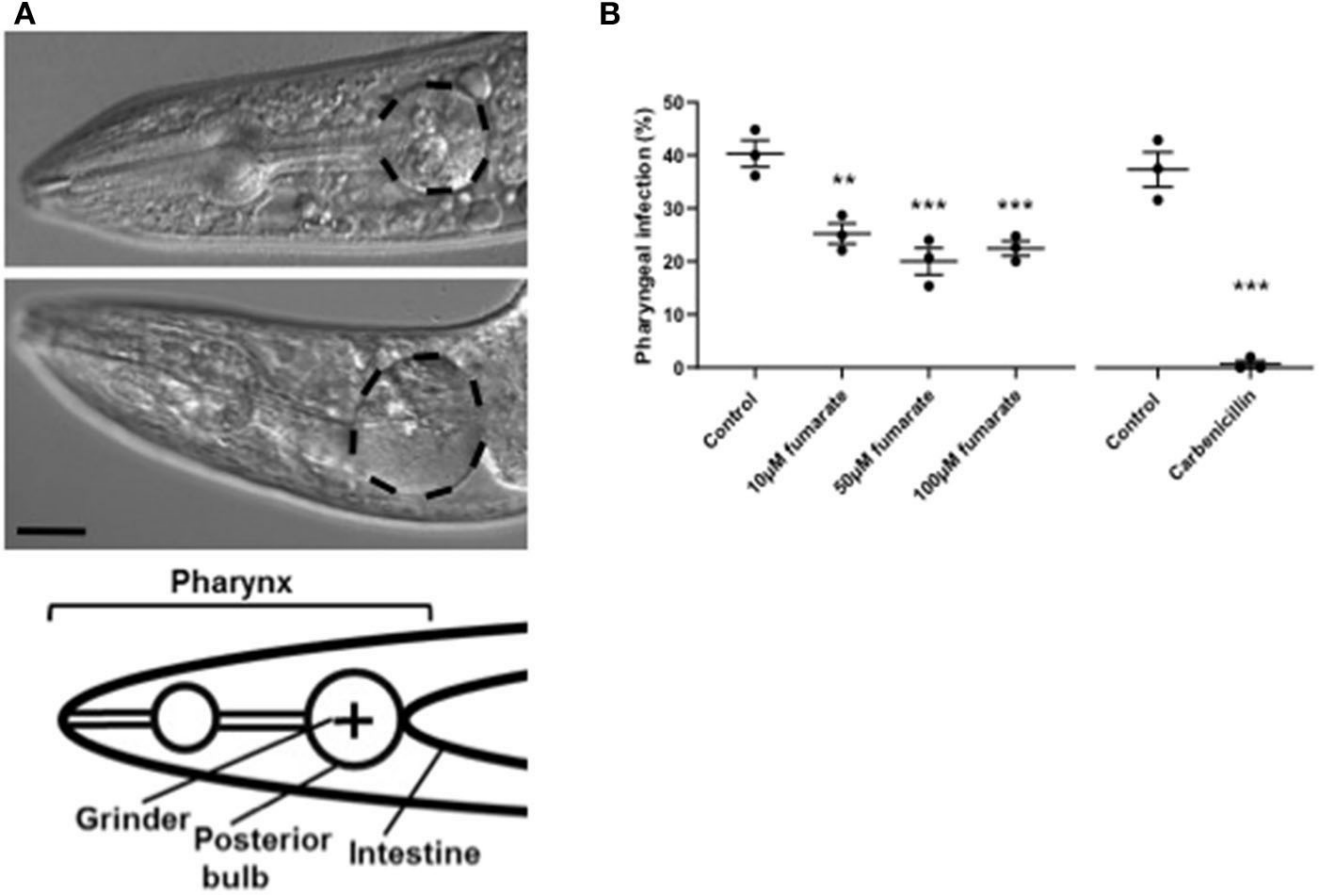
Fumarate reduces pharyngeal infection in *C. elegans*. **(A)** DIC images of *C. elegans* pharynx, at day 13 of worm adulthood, showing infection (top); and no infection (middle); the posterior bulb is outlined (scale bar = 40 μm), and a graphic representation (bottom). **(B)** Percentage of *C. elegans* with pharyngeal infection in un-treated (control), and 10, 50, and 100 μM MMF-treated worms, as well as in *C. elegans* cultured on carbenicillin (4 mM)-treated *E. coli* OP50. Each dot represents a trial performed by triplicate, and in each triplicate about 50 worms were analyzed. Bars depict mean ± s.d. (*n* = 3). **p < 0.01, ***p < 0.001, by one-way analysis of variance and unpaired Student's *t*-tests.

Around 40% of aging *C. elegans* adults die from life-limiting bacterial pharyngeal infection, under standard culture conditions (*E. coli* OP50, 20°C) (30). To explore whether fumarate plays a role in the *C. elegans* innate immune response, we investigated the effects of MMF on pharyngeal infection. Fumarate supplementation was found to cause a statistically significant 50% reduction in incidence of death with pharyngeal infection (Figure 7), at concentrations of 10, 50 and 100 μM. The effect showed a higher degree of statistical significance at 50 and 100 μM, suggesting a possible dose-dependent effect (though the difference between the effects of 10 and 50 μM or 100 μM were not significant).

## Discussion

Innate immune training offers a promising theoretical framework for the understanding of infectious diseases and vaccination (31–34), and mechanistically, it has been defined in terms of immunological, metabolic and epigenetic hallmarks (2, 31, 35–38). This work analyzed some mitochondrial traits during the induction phase of MMF-induced innate immune training and showed distinctive features of mitochondrial function, from the first minutes of fumarate addition to monocytes, helping to define a mitochondrial signature for innate immune training.

Fumarate is an intermediate metabolite in the tricarboxylic acid cycle produced within mitochondria (18, 39), which accumulates in β-glucan- but not in BCG-trained monocytes and is able, by itself, to induce innate immune training in monocytes (12).

It was first confirmed that MMF induces training in monocytes, as demonstrated by Arts et al. (12) i.e., after MMF treatment and seven days of “resting,” LPS stimulation of monocytes/macrophages resulted in higher production of TNF-α, as compared to untreated cells (Figure 1). When monocytes were trained with heat-killed *C. albicans*, as shown by Quintin et al. (6), a more robust production of TNF-α was observed upon LPS stimulation, as compared to fumarate-trained monocytes (Figure 1).

From the several known inducers of innate immune training (5–10, 12), we decided to assess the mitochondrial signature on fumarate-induced training, reasoning that if the induction of innate immune training is to be used for better vaccination protocols, the use of a simple molecule with several well-defined biological activities, such as fumarate, would have advantages over the use of whole microorganisms, such as BCG, or microorganism-derived products, β-glucan for instance.

Fumarate is produced by the oxidation of succinate by the enzyme succinate dehydrogenase (respiratory complex II, CII) within mitochondria (40), and it is also a product of tyrosine metabolism and the urea and purine nucleotide cycles (24, 41). In addition to its function as a TCA cycle metabolic intermediate, fumarate has antioxidant, epigenetic, and immune response modulation functions (12, 41–45).

Next, considering that mitochondrial calcium shapes Ca^2+^ signaling and stimulates respiration and ATP synthesis (21, 46) both mitochondrial and cytoplasmic calcium fluxes were assessed. Results showed that mitochondria readily respond to stimulation with MMF by uptaking calcium within minutes (Figures 2B,D), suggesting that MMF binds to a fumarate receptor in the cell membrane, triggering cytoplasmic calcium influx (Figures 2A,C) followed by mitochondrial buffering (47). In this regard, a fumarate receptor, the hydroxycarboxylic acid receptor 2 (HCAR2), has been described (48). However, when mitochondrial calcium was assessed in the absence of extracellular calcium, calcium influx into mitochondria was still observed (Figure 2E), thus suggesting that fumarate can also trigger the release of calcium from intracellular stores, allowing calcium influx into mitochondria. Endoplasmic reticulum (ER)-mitochondria tethering and Ca^2+^ transfer to the mitochondrial matrix via ER–mitochondria contact sites is a well-known mechanism of cellular calcium handling (49, 50); whether this mechanism accounts for fumarate-induced innate immune training would require further analyses.

Mitochondria also responded to the fumarate treatment of monocytes by driving their mitochondrial dynamics toward a fusion state and by increasing, within a few hours, their membrane potential (Δψm) (Figure 3). At the ultrastructural level, mitochondria became larger, as assessed by the morphological parameters described by Picard et al. (27) i.e., surface area, perimeter, Feret diameter, aspect ratio, and form factor, whereas roundness and circularity decreased, as compared to untreated cells (Figure 4). These ultrastructural characteristics are compatible with the finding of mitochondrial fusion (Figure 3); increased fusion may be a requirement to maximize oxidative phosphorylation by means of complementation among mitochondria, and to maintain the energy output in the face of stress (22).

In addition to mitochondrial dynamics, cristae are also an important morphological indicator of mitochondrial function since cristae are the hub where most of the respiratory complexes are embedded, and account for oxidative phosphorylation and ATP production and therefore changes in cristae number and shape define not only respiratory capacity but cell viability as well (51). Mitochondrial cristae structure has been defined in terms of density (cristae number vs. mitochondrial area), and by the incident angle, which indicates how closely cristae are arranged; the lower the incident angle is, the closer cristae are to each other, making the respiratory electron transfer chain more efficient (28). Results showed that the mitochondrial incident angle was lower in fumarate-treated monocytes as compared to that of untreated monocytes (Figure 5), suggesting that fumarate could favor the assembly of respiratory chain supercomplexes (23, 51, 52), and this remains to be analyzed.

In addition to analyzing the effect of MMF on the mitochondria of human PBMC-derived monocytes in the context of innate immune training, we wanted to explore its effects *in vivo* in a whole organism. To this end, two strains of *C. elegans* were used: SJ4103 (GFP-labeled mitochondria in muscle) and SJ4143 (GFP-labeled mitochondria in intestinal cells). Both strains of *C. elegans* showed an effect of MMF on mitochondrial dynamics similar to that in monocytes. This indicates that MMF-induced mitochondrial fusion is not exclusive to human monocytes, and suggest that the effect is widely conserved in the animal kingdom.

Recently, it has also been shown that nutrient deficiency induces mitochondrial fusion in *C. elegans*, and that this correlates with resistance to *Enterococcus faecalis* infection (53). We tested whether fumarate (which induces mitochondrial fusion) can lower the risk of infection in *C. elegans*. Under standard culture conditions on *E. coli* OP50, *C. elegans* is susceptible to life-limiting pharyngeal infection that begins in the terminal bulb around the grinder and progresses to the rest of the pharynx (30). We found that in MMF-treated *C. elegans*, incidence of death with pharyngeal infection incidence drops by 50% (from ~40% incidence in untreated to ~20% in MMF-treated worms), at a MMF concentration of 50 μM. Since it has been shown that fumaric acid, and dimethyl fumarate affect bacterial growth at a concentration of 10 mM (i.e., 200 times this concentration) (39), we cultured *E. coli* OP50 in the presence of 100 and 500 μM MMF, the form of fumarate used in all experiments, and could detect no effect on bacterial growth (data not shown), thus arguing against the possibility that the observed increased resistance of *C. elegans* to infection is due to effects on bacterial viability. However, a mild impairment of E. coli pathogenicity by fumarate affecting pharyngeal infection rate cannot be ruled out.

This work provides evidence for a mitochondrial signature, consisting of the calcium influx to cytoplasm and mitochondria within a few minutes, and the fusion, polarization, and increase in mitochondrial cristae closeness, within a few hours post-stimuli, suggesting mitochondrial activation, and highlighting the mitochondrial side of the metabolic basis for the induction phase of fumarate-induced innate immune training. Moreover, results showed that MMF induces mitochondrial fusion in *C. elegans*, which were accompanied by *C. elegans* resistance to pharyngeal infection under standard culture conditions. How specific these mitochondrial traits are for pro-inflammatory as compared to anti-inflammatory innate training remains to be analyzed (6, 54).

A further caveat is that *E. coli* is not a pathogen that *C. elegans* usually encounters in the wild (55); however, given that *C. elegans* immunity has presumably evolved to deal with highly diverse and sometimes novel bacterial pathogens, the observed effect of fumarate in its response to *E. coli* is likely to reflect an authentic immunological mechanism.

Together, these findings open the possibility to experimentally modulate mitochondrial activity to boost innate immune training and resistance to infection, and illustrate the potential for using *C. elegans* as a whole organism model of innate immune training.

## Data Availability Statement

The raw data supporting the conclusions of this article will be made available by the authors, without undue reservation.

## Ethics Statement

The studies were reviewed and approved (LSHTM-5520 and LSHTM-14576). All healthy blood donors provided their written informed consent to participate in this study.

## Author Contributions

All authors listed have made a substantial, direct and intellectual contribution to the work, and approved it for publication.

## Conflict of Interest

The authors declare that the research was conducted in the absence of any commercial or financial relationships that could be construed as a potential conflict of interest. The reviewer RA declared a past co-authorship with one of the authors HD to the handling Editor.
